# Arsenic-Doped High-Resistivity-Silicon Epitaxial Layers for Integrating Low-Capacitance Diodes

**DOI:** 10.3390/ma4122092

**Published:** 2011-12-06

**Authors:** Agata Sakic, Tom L. M. Scholtes, Wiebe de Boer, Negin Golshani, Jaber Derakhshandeh, Lis K. Nanver

**Affiliations:** ECTM-DIMES, Delft University of Technology, Feldmannweg 17, Delft 2628CT, The Netherlands; E-Mails: t.l.m.scholtes@tudelft.nl (T.L.M.S.); w.b.deboer@tudelft.nl (W.B.); n.golshani@tudelft.nl (N.G.); j.derakhshandeh@tudelft.nl (J.D.); l.k.nanver@tudelft.nl (L.K.N.)

**Keywords:** silicon epitaxy, high-resistivity epi-layers, arsenic auto-doping, arsenic segregation, charged-particle detection, diode capacitance, photodiodes

## Abstract

An arsenic doping technique for depositing up to 40-μm-thick high-resistivity layers is presented for fabricating diodes with low RC constants that can be integrated in closely-packed configurations. The doping of the as-grown epi-layers is controlled down to 5 × 10^11^ cm^−3^, a value that is solely limited by the cleanness of the epitaxial reactor chamber. To ensure such a low doping concentration, first an As-doped Si seed layer is grown with a concentration of 10^16^ to 10^17^ cm^−3^, after which the dopant gas arsine is turned off and a thick lightly-doped epi-layer is deposited. The final doping in the thick epi-layer relies on the segregation and incorporation of As from the seed layer, and it also depends on the final thickness of the layer, and the exact growth cycles. The obtained epi-layers exhibit a low density of stacking faults, an over-the-wafer doping uniformity of 3.6%, and a lifetime of generated carriers of more than 2.5 ms. Furthermore, the implementation of a segmented photodiode electron detector is demonstrated, featuring a 30 pF capacitance and a 90 Ω series resistance for a 7.6 mm^2^ anode area.

## 1. Introduction

Recent advances in silicon photodiodes have yielded detectors with near-theoretical detection efficiencies and fast response times [1,2,3]. The latter is determined by RC constant, *i.e.*, the series resistance R and junction capacitance C of the photodiodes comprising the detector. In some cases the Si-substrate is used as the one terminal of the photodiode, in which case the substrate doping can be decisive for the overall RC. The wider the depletion over the diode junction, *i.e.*, the lower the doping, the lower the C, and the higher the doping is in the non-depleted region, the lower the R. Therefore, a trade-off should be made when choosing the doping level assuming it is uniform through the substrate. One solution is to use high-resistivity substrates that can be fully-depleted, as has been demonstrated for single photodiode segment X-ray detectors [4]. The lateral spread of the depletion is then about the same size as the thickness of the wafer which is usually greater than ~250 μm. This means that for multi-segment detectors the individual photodiodes should be electrically isolated by a distance that is much greater than this. However, modern solid-state detectors demand flexible multi-segmentation (pixelization) of the photosensitive surface with closely-packed segments [5]. This implies that their depletion region has to be vertically wide to obtain the low capacitance and series resistance, but laterally limited in order for neighboring detector segments (adjacent diodes) to function independently. One solution is to fabricate detectors on high resistivity, high-quality, thick epitaxial layers, grown on low-to-medium resistivity substrates, thus providing wide depletion, lateral isolation, and low series resistance through the substrate.

The use of epitaxially grown, thick, high-resistivity Si layers for radiation detection has been investigated in the past for the ability to enhance the radiation hardness at high radiation fluences [6,7]. Layers were grown >100-μm-thick with a resistivity of a few kΩcm that contained a high concentration of deep level traps. These traps served as “sinks” for radiation induced interstitial defects, minimizing the radiation-induced damage of the detector. As a drawback the dark current is increased to a level that is unacceptable for many applications such as the low-energy electron detectors that have motivated the work presented in this paper. These detectors were developed for use in advanced Scanning Electron Microscope (SEM) systems. The required speed translated into an R of less than 100 Ω and a C of less than 5 pF/mm^2^ can be achieved with a depletion width over the photodiode junctions of about 30 µm. To maintain a separation of segments of ~100 μm, this requires a doping of 10^12^–10^13^ cm^−3^.

In the present work, up to 40-μm-thick high-quality defect-free epi-layers are grown in a commercial Si/SiGe chemical-vapor-deposition (CVD) epitaxial reactor system. For achieving the required light doping levels, doping from arsine rather than phosphine is preferred because of the much higher incorporation levels of phosphorus. However, using arsine is challenging due to its strong tendency to segregate on the surface and subsequent auto-doping behavior. Past work has shown that these properties can be controlled and applied with benefit to create special doping profiles [8,9]. Here a technique is developed for a controlled segregation, removal and auto-doping of As to obtain the required very lowly-doped profiles.

## 2. Fabrication

### 2.1. As Doping

The growth of the n-type Si epi-layer is carried out in an RPCVD Epsilon One reactor using dichlorosilane (DCS) and arsine (AsH_3_) as precursors for Si and As, respectively. As a first step a “seed” layer of As-doped Si is grown on the wafer, from which the As atoms segregate on the seed surface and are removed by a high temperature baking step (desorption step) at 1,100 °C. After that, the thick epi-layer is grown in several steps with only the DCS turned on, performing a desorption bake after each step to remove the As from the surface**.** The doping concentration and the doping profile of the obtained epi-layers are determined by As segregation where, depending on the temperature of the deposition, only a fraction of segregated As atoms gets incorporated into the as-grown layer, while the rest remains on the layer surface [10]. The final doping level in the Si, *N_d_(x)* can be calculated as

(1) $$N_{d}\left(x\right)\;=\;i_{R}N_{Si}\theta \left(t\right)$$

where *N_Si_* is the Si concentration of 5 × 10^22^ cm^−3^, *i_R_* the incorporation rate, and *θ(t)* the percentage of a full As monolayer, *N_ML_* = 6.8 × 10^14^ cm^−2^, covering the surface. While the incorporation rate depends mainly on the solid solubility of the As atoms in Si at the defined deposition temperature, the surface coverage *θ(t)* is governed by the mass balance of the following four processes: adsorption of the As atoms on the surface (A), segregation (S), desorption from the surface (D), and incorporation into the epi-layer (I):

(2) θ(t)=A + S−I−D

**Figure 1:**
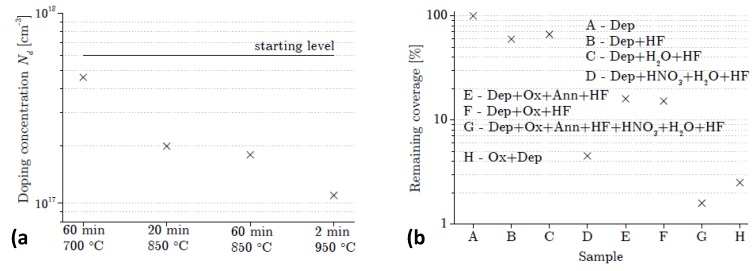
(**a**) Reduction in doping level after baking the sample at different temperatures; (**b**) Reduction of the As surface coverage with different ex-situ chemical cleaning steps.

To obtain low doping concentrations, the surface coverage *θ(t)* should be low, and therefore the adsorption of the As atoms needs to be minimized and the desorption maximized. The surface concentration is then restricted by the segregation and incorporation rates and their mutual interactions. To minimize adsorption, the dopant gas AsH_3_ is turned off during the growth of the low-doped epi-layer. Moreover, the desorption of the segregated atoms is enhanced by applying a high-temperature baking step prior to each new growth cycle. The desorption baking step has been characterized in several studies of situations where it is important to have precise control of the As covering the Si (100) surface [11,12,13]. Some of the results are shown in Figure 1(a) where it can be seen that there is a strong temperature dependence of the desorption, while the effect saturates in time so that 20 min or 60 min at 850 °C give practically the same result. The 2 min, 1,100 °C bake step used in this work is commonly used, also in combination with high-dose As-implanted buried layers, to reduce the As surface coverage. The residual As segregated on the surface after the baking step supplies As for incorporation during the subsequent Si-epi growth, which is essentially an auto-doping mechanism. The segregated As atoms which neither desorb from the surface nor incorporate in the Si remain at the top surface of the epi-layer in a float-like manner, or more precisely, Si dangling bonds are replaced by lone pairs of As electrons. At the end of a growth sequence, the residual segregated As on the wafer surface can be removed chemically. The desorption efficiency as a function of chemical processing is illustrated in the Figure 1(b) [11]. This is done here by cleaning successively in HNO_3_, H_2_O, and HF before further processing such as the deposition of the PureB anode region. When the supply of arsine is turned off, and an initial As coverage that gives an incorporated doping of Nd(0) is present, then equation (1) can be reduced to describe the doping from further incorporation from the remaining As coverage as an exponentially decreasing profile:

(3) $$N_{d}\left(x\right)\;=\;N_{d}\left(0\right)exp(-i_{R}N_{Si}x/N_{ML})$$

where L = *N_ML_/i_R_N_Si_* is the characteristic length which describes the incorporation rate at the given process temperature [11].

Since the overall doping level is very low, the resulting doping may be influenced by the background doping in the chamber which will set the minimal obtainable doping level. Therefore, it is vital to thoroughly clean the chamber before deposition. The growth of the seed layer itself may also contribute to the chamber background level. The final profile of the first few micron of the epi is dominated by up-diffusion of the As. Since the growth rate is around 1 μm/min, and layers as thick as 40 μm are grown, we can assume that the up-diffusion of As from the seed layer can be up to 10 μm when the growth temperature of 1,050 °C and the bake steps at 1,100 °C are taken into account.

### 2.2. Thick High-Resistivity Si Epi-Layer Growth

Experiments are performed using n-type (100) 2–5 Ω cm Si substrates. They are dip etched for 4 min in HF 0.5%, Marangoni dried just before the epitaxy, and in the epitaxial reactor itself the wafers are baked at 1,100 °C to ensure a native oxide free surface. A 300-nm-thick As-doped seed layer is then grown at 1,050 °C with a doping concentration of either 10^16^ or 10^17^ cm^−3^. The seed doping level has to be both low enough to produce the low capacitance values, and high enough to prevent the counter-doping phenomenon. The trade-offs for the resulting doping profiles and capacitances for both 10^16^ or 10^17^ cm^−3^ will be discussed in the following sections. The seed layer formation is followed by a desorption step at 1,100 °C to remove the As atoms that have segregated on the surface. The arsine is turned off and a series of Si layers are grown at 1,050 °C to a thickness of 5 μm, 10 μm, 20 μm, and 40 μm. The latter two thicker layers of 20 μm and 40 μm may be grown in steps of 10 or 20 μm, to lower the risk of excessive deposition on the chamber walls during the long growth cycles involved.

### 2.3. Photodiode Fabrication

**Figure 2:**
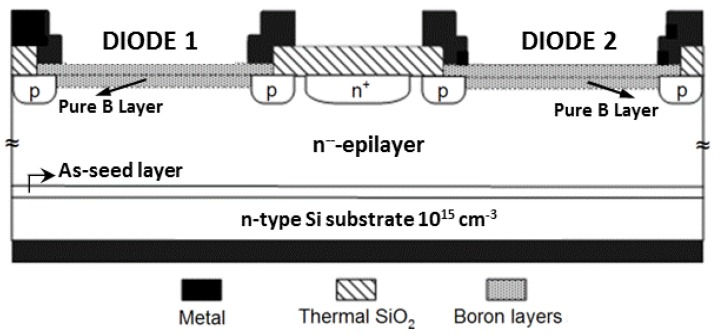
Schematic cross-section of two adjacent PureB diodes fabricated on a low-doped epi-layer wafer.

The thick epi-layer substrates are used for fabricating sets of photodiodes with low capacitance. A schematic of two adjacent diodes is presented in Figure 2. First, the wafers are cleaned as described in the previous section to remove native oxide and reduce the As coverage on the surface. Then 200 nm of the thermal oxide is grown through which n^+^- and p-type guard rings are implanted. After annealing the implantations, LPCVD oxide is deposited and finally the oxide (both thermal and LPCVD) is patterned and etched, to form the anode openings of the photodiodes. The anode area used in the present work is 7.6 mm^2^. The anodes are then deposited in an RPCVD reactor using the recently developed PureB technology, parameters of which are described in [14,15]. In this technology a nanometer-thin amorphous pure boron layer is selectively deposited on an oxide-free Si surface at 700 °C to form an ultrashallow junction that is reliable both electrically and optically [2]. Finally, metallization layers are deposited on the front and back of the wafer and patterned to form the anode contact pads and the cathode contact on the back of the wafer.

## 3. Profile Engineering

When a high resistivity epi-layer is grown as described in the previous section, with a set temperature and pressure, the doping profile is essentially determined by three parameters: the total thickness of the epi-layer, the doping concentration of the seed layer, and the number of steps used to grow the lightly-doped layer. In this section each parameter will be separately studied with respect to the resulting doping profile which is measured by C-V profiling [16]. The carrier density N(W) and the space charge region width (W) are extracted from the C-V characteristics as follows:

(4) $$N(W)=-\frac{C^{3}}{q\varepsilon_{o}\varepsilon_{s}A^{2}dC/dV}=\frac{2}{q\varepsilon_{o}\varepsilon_{s}A^{2}d\left(1/C^{2}\right)/dV}$$

(5) $$W=\frac{\varepsilon_{o}\varepsilon_{s}A}{C}$$

The C-V curve was measured with a capacitance meter set to series connection measurement at 1 MHz. According to [16], the instrumentation error in series connection can be calculated as

(6) $$\%\;error=0.1\sqrt{1+{\left(2\pi fC_{S}R_{S}\right)}^{2}}$$

The typical values of capacitance and resistance measured in this work are ~100 pF and ~100 Ω, giving an instrumentation error of only 0.1%. The capacitance meter has a provision for the cable-length and contact corrections that were implemented prior to measuring.

In all the reported figures showing doping profiles, 0 depth on the x-axis corresponds to the upper surface of the detector where the detector anode is placed. Since the p^+^-region is only a few nm deep, the depletion into this region can be set at 0 μm without loss of profiling accuracy. The left y-axis displays the carrier density, and the right y-axis the reverse voltage value at which the depletion width (x-axis) and the doping density (left-y-axis) are obtained. In the extraction of the profile it is assumed that parasitic capacitances can be neglected in view of the large diode area. These parasitics include the diode perimeter capacitance and the MOS capacitance under the tracks and pads connecting the diode. An extensive simulation analysis of the perimeter effects of PureB diodes has been made and is in part published in [17], where it is found that for the present device structure the bulk capacitance is 3.52 × 10^−18^ F/μm^2^ while the perimeter capacitance is 1.1 × 10^−16^ F/μm. For the toroidal-shaped diode measured here with an area of 7.6 mm^2^, and a perimeter of 23 mm, the bulk capacitance is then 26.7 pF and the perimeter capacitance is only 2.53 pF. This ~10% contribution of the perimeter will have some influence on the value of the extracted capacitance but, relatively, it will result in higher doping the higher the actual doping is. Therefore, the comparisons that are made remain valid. For an entirely correct extraction of the doping profiles, a differential C-V measurement should be made to completely eliminate perimeter effects.

One more correction that has to be taken into account for very low doping concentrations that drop below 10^12^ cm^−3^ is the depletion region at 0 V bias that already penetrates the space charge region for more than 15 μm. Thus, a small forward voltage (up to 0.3 V) is required to profile the dopants in the upper parts of the epi-layer. At a certain voltage, measured here around 0.15 V, the diffusion capacitance starts affecting the measurement results, giving a non-physical increase in the doping concentration of the layer that will be disregarded.

### 3.1. Epi-Layer Thickness

**Figure 3:**
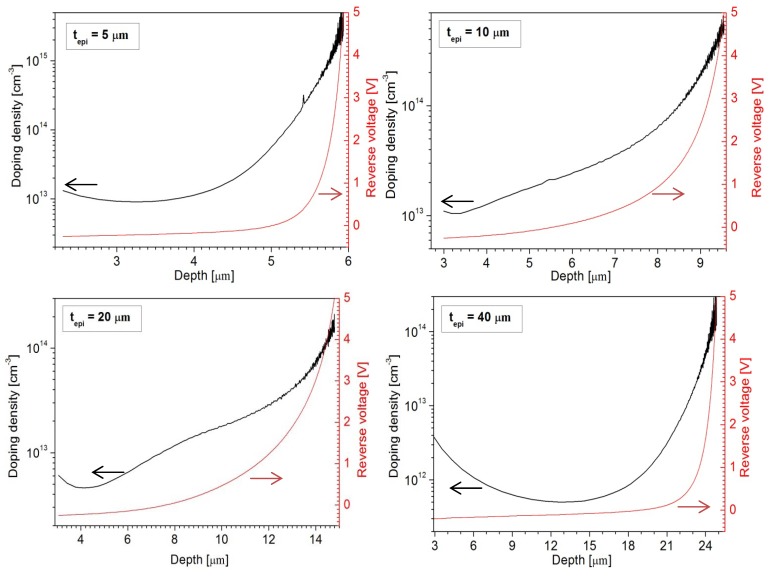
CV-doping profiles and reverse bias as a function of depletion width for As-doped epitaxial layers grow to a thickness of 5 μm, 10 μm, 20 μm, and 40 μm.

**Figure 4:**
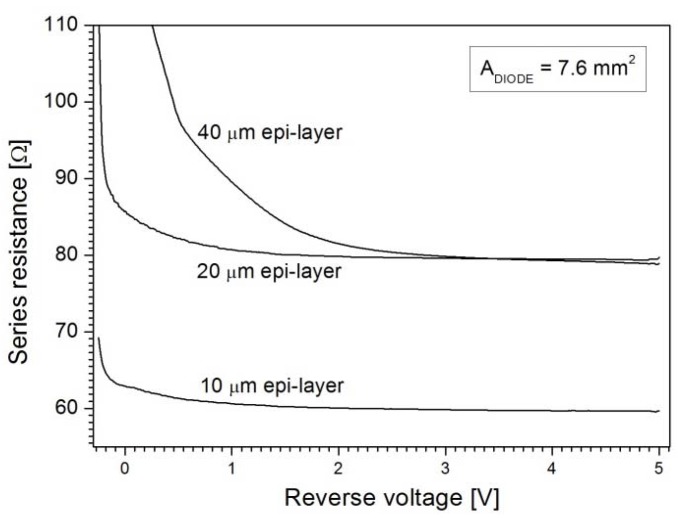
Series resistance of the 7.6 mm^2^-area diode for 10 μm, 20 μm, and 40 μm epi-layer thicknesses displaying the influence of the undepleted remnant of the epi-layer.

**Figure 5:**
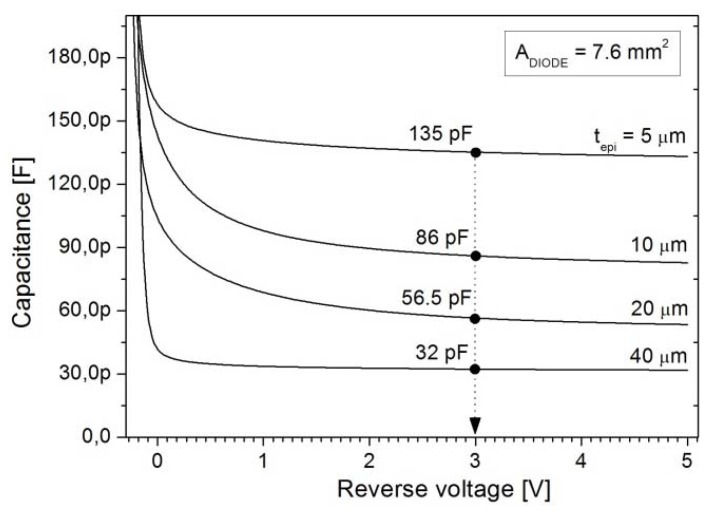
Capacitance measurements for photodiodes with 7.6 mm^2^ anode area fabricated on the substrates characterized in Figure 2.

The choice of the epi-layer thickness is determined by the application. In this work, the requirements were set by the application of the photodiodes in high-responsivity backscattered-electron detectors used in SEM systems. In such systems, the electron energies typically range up to 30 keV at which the electrons will penetrate the Si bulk no more than 10 μm [18]. Hence, an epi-layer that can be depleted to 10 μm at the operating voltage needs to be fabricated. However, the capacitance specification of <5 pF/cm^2^ dictates a much thicker depletion width. Figure 3 shows the doping profiles of four epi-layers of thickness 5 μm, 10 μm, 20 μm, and 40 μm. It can be seen that the depletion widths at an operating voltage of 3 V reverse bias are around 6 μm, 9 μm, 14 μm, and 25 μm, respectively. Even if the 5 μm, 10 μm, and 20 μm layers were to be fully depleted by a higher voltage, only the 40 μm thick epi-layer would meet the specification of <5 pF/cm^2^. The undepleted part of the layer must also be taken into account because it can be decisive for the series resistance. For example, the 20 μm and 40 μm epi-layers are not fully depleted at 3 V and the undepleted region will contribute to the series resistance of the photodiodes. As demonstrated by the graphs in Figure 4 the series resistance is about 20 Ω higher than for the 10-µm-epi case at voltages higher than 3 V, while it increases dramatically as the voltage goes to lower values at which the undepleted region is wider. Therefore, the epi-layer thickness should be as close as possible to the targeted depletion thickness for an optimal trade-off between capacitance and resistance. The respective capacitance values measured at 3 V are 135 pF, 86 pF, 56.5 pF, and 32 pF, as indicated in Figure 5.

### 3.2. As Seed-Layer Doping

**Figure 6:**
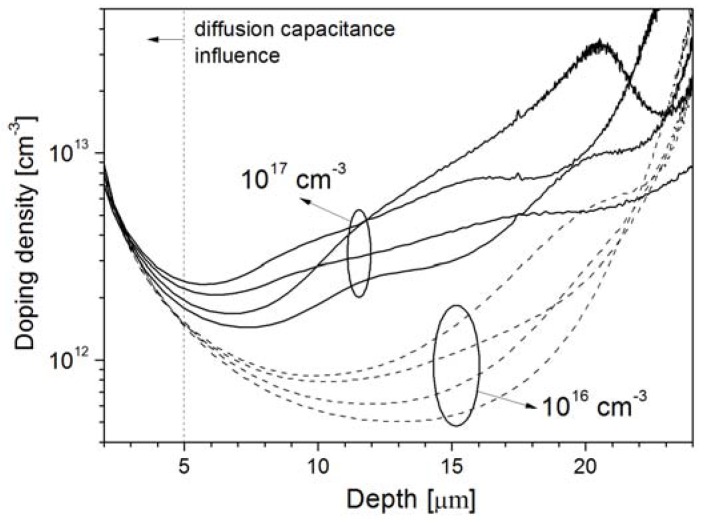
CV-doping profiles of 40-μm-thick epi-layers with 10^16^ cm^−3^ and 10^17^ cm^−3^ As-seed layer concentrations. The doping is profiled up to 5 V reverse bias (W = 25 μm).

Since the growing of the As-doped seed layer defines the As-segregated surface doping and the background doping level of the chamber, its concentration is decisive for the doping of the lightly-doped epi. In the example shown in Figure 6, several different 40-μm-thick substrates with seed layer concentrations of 10^16^ and 10^17^ cm^−3^ have been profiled.

**Figure 7:**
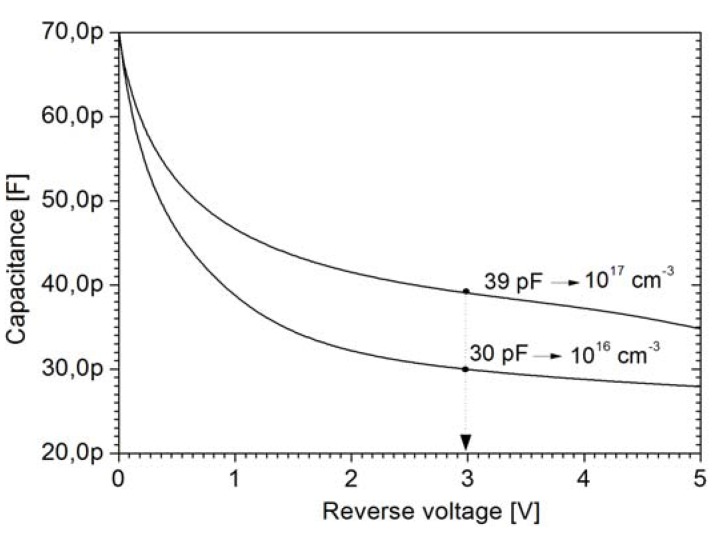
Diode capacitance for 40-μm-thick epi-layers with As-seed layer concentrations of 10^16^ cm^−3^ and 10^17^ cm^−3^, for 7.6 mm^2^ anode areas.

The samples with a 10^17^ cm^−3^ seed-layer exhibit profiles which are up to an order of magnitude higher in concentration than the samples with the 10^16^ cm^−3^ seed-layers, as indicated in Figure 6. For example, the doping density for the 10^17^ cm^−3^ seed-layer sample at the depth of 10 μm is 4 × 10^13^ cm^−3^, while for the 10^16^ cm^−3^ sample it drops to 6 × 10^12^ cm^−3^. Correspondingly, the voltage required to deplete the first 10 μm of the substrate differs around 0.5 V in value. This causes a spread of capacitance values at 3 V operating voltage of up to 30%, ranging from 30 pF to 40 pF as shown in Figure 7.

### 3.3. Growth Cycles

**Figure 8:**
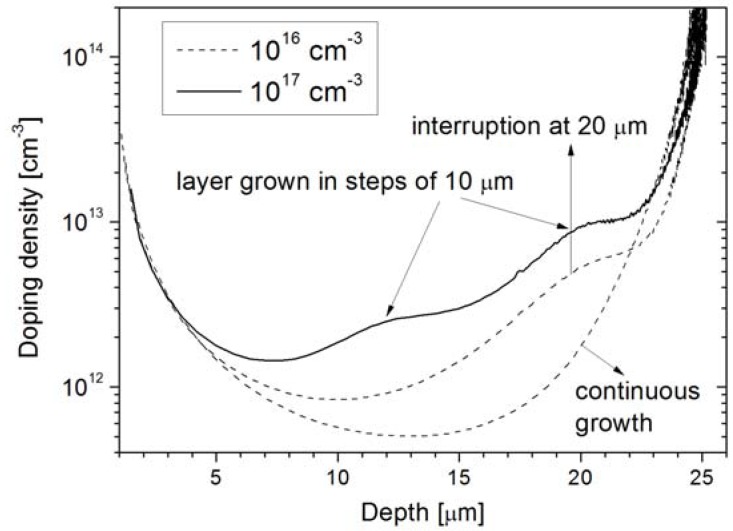
CV-doping profiles for 40-μm-thick layer grown either in one go, in 10 μm steps, or 20 μm steps. The doping is profiled up to 5 V reverse bias (W = 25 μm).

Besides the differences in the doping level of epi-layers related to different seed-layer concentrations, non-uniformities in the layer doping are also detected when different growth cycles are introduced. In Figure 8 three distinct 40-μm profiles are shown, each grown in a different manner: in one go, in steps of 10 μm, or steps of 20 μm. With the 10-μm steps, bumps of increase in the doping are seen at about 10 μm and 20 μm, as marked in the figure. Likewise, for the 20-μm steps a bump is seen at about 20 μm. The increase in doping can be caused by a combination of increased segregation of As on the surface during the 1,100 °C baking step, as well as doping from the background contamination of the chamber. The shape of the bumps is determined by the total temperature processing. Both effects will be stronger when a high seed-layer doping is implemented. In the following Si growth that proceeds at 1,050 °C, the bump doping decreases in accordance with the fact that the As surface concentration is slowly built in, and at the same time the background level in the chamber is also slowly consumed.

**Figure 9:**
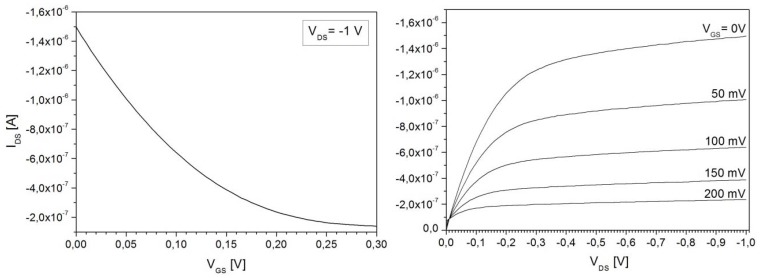
Transfer and output characteristics of the parasitic JFET between two adjacent diodes caused by p-type counter-doping of the n-epi.

**Figure 10:**
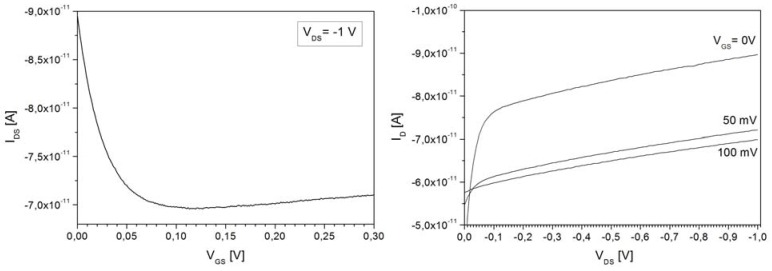
Transfer and output characteristics between two adjacent diodes that are well isolated, *i.e.*, have no p-type counter-doping of the n-epi.

Comparing the two 10^16^ cm^−3^ seed-layer samples, it can be seen that the doping density significantly depends on whether the layer was grown continuously, or with the interruptions. In the vicinity of the bump at 20 μm depth, the doping value rises from around 2 × 10^12^ cm^−3^ to 5.5 × 10^12^ cm^−3^ and further on remains slightly higher throughout the whole layer. The growth that is carried out in steps is favorable in cases when the epi-layer doping level drops low, typically at the end of the growth sequence as the available As doping gets consumed. This increases the danger of p-type counter-doping due to chamber contamination. In cases where such unintentional p-type counter-doping was encountered it was impossible to perform C-V profiling because the diode is not well isolated from the rest of the wafer and very high leakage currents result. However, the p-doping can be identified by connecting two adjacent diodes shown in Figure 2 in a JFET operation mode where the two anodes function as source/drain and the cathode functions as a gate to modulate the channel. An example of such a measurement is shown in Figure 9 where the output and transfer characteristics show a clear JFET-like behavior, with a pinch-off voltage of about 0.2 V. The pinch-off voltage and the on-currents are much higher than the ideal case without p-type counter-doping. In Figure 10 the measurement is shown as performed on well-isolated adjacent diodes and it can be seen that the current levels correspond to leakage current levels of about 50 pA of the diodes themselves.

## 4. Quality of the Epi-Layers

The quality of the epi-layers studied here is evaluated using three parameters: the stacking fault density, the level of the dark current of the fabricated diodes, and doping uniformity across the wafer. The density of stacking faults depends on the quality of the starting substrate, but for low levels these faults do not impact the final performance of the mm^2^-large diodes as reported in [19]. The dark current level reflects the number of active generation-recombination centers in the epi-layer, and the doping uniformity is a measure of the reliability of the growth method.

**Figure 11:**
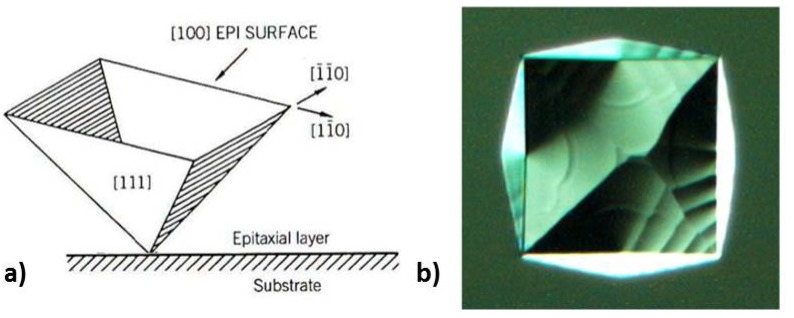
(**a**) Stacking fault geometry on (100) silicon [20]; (**b**) SEM image of a stacking fault grown in a silicon epitaxial layer of 40 µm thickness.

**Figure 12:**
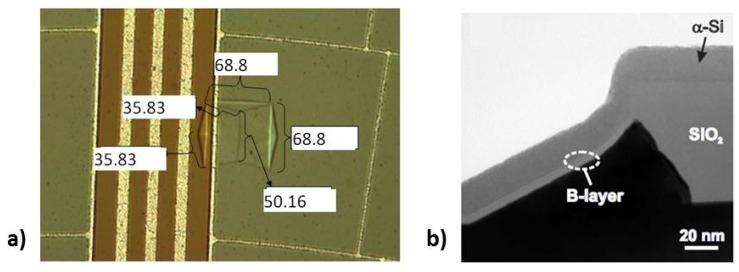
(**a**) Optical image of a stacking fault at the edge of a photodiode surface showing neighboring metal tracks and dimensions in microns, epi-thickness 40 µm. (**b**) TEM image showing conformal growth of a PureB layer [14].

Stacking faults are pyramid-shaped crystallographic defects caused by a lattice mismatch at the surface of the substrate, or during the growth. Typical geometries are shown in Figure 11. The final size which is observed on the wafer surface depends on the depth of the first disruption that initiated the stacking fault, and is reported up to be around 70 × 70 μm^2^ in this work for 40-µm-thick layers (Figure 12(a)). Despite the size, for the PureB diodes a limited number of such defects does not affect the performance in terms of dark current, capacitance, and optical responsivity of the photodiodes. This is because the diodes are large and the PureB technology provides conformal coverage of the anode surface even when deviating crystallographic orientations are present, as shown in Figure 12(b) [14]. The density of the stacking faults is mainly counted to be about 2 per cm^2^.

**Figure 13:**
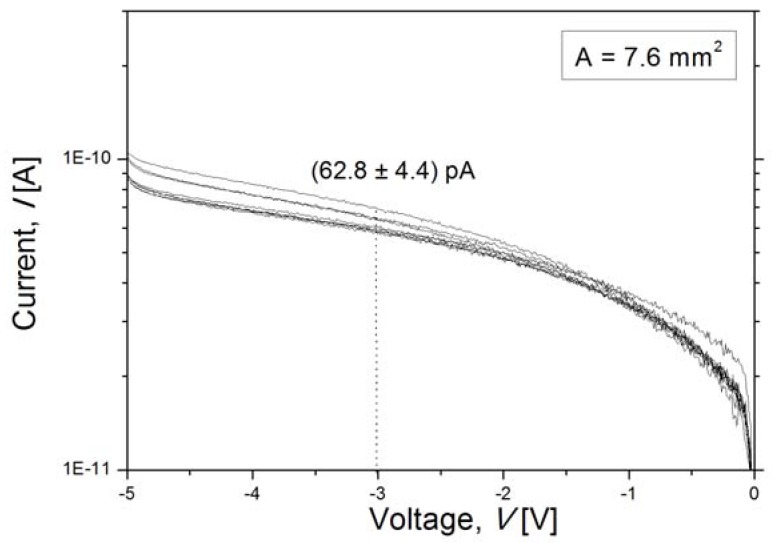
Reverse current of a PureB photodiode fabricated on a 40 μm thick epi-layer.

The dark current is measured on several diodes fabricated on the 40-μm-thick epi-layer and exhibits a very low current level measured up to 5 V reverse bias with a small spread over the wafer as seen in Figure 13. The average measured dark current is 63 pA at 3 V reverse bias and consists of bulk and surface generation-recombination currents as well as the diffusion current component. If, in the worst case, all leakage current is dominated by the bulk generation current, assuming the complete suppression of the surface generation current and a negligible diffusion current, it could be calculated as

(7) $$I_{bulk}=q\frac{n_{i}}{2\tau_{g}}AW_{depl}$$

where *q* is the charge unit, *n_i_* is the intrinsic carrier concentration, τ_g_ is the generation lifetime, *A* is the anode area of the detector of 7.6 mm^2^, and *W_depl_* is the width of the depletion region of 25 μm at 3 V reverse bias (Figure 13). Then the generation lifetime is estimated to be ~2.5ms. Moreover, due to the neglected leakage current components, it is safe to claim that the generation lifetime for the 40-μm-thick epi-layer is >2.5 ms. This is more than an order of magnitude higher than the ~100 μs lifetime of the thick, high-resistivity epi-layers reported in [6].

**Figure 14:**
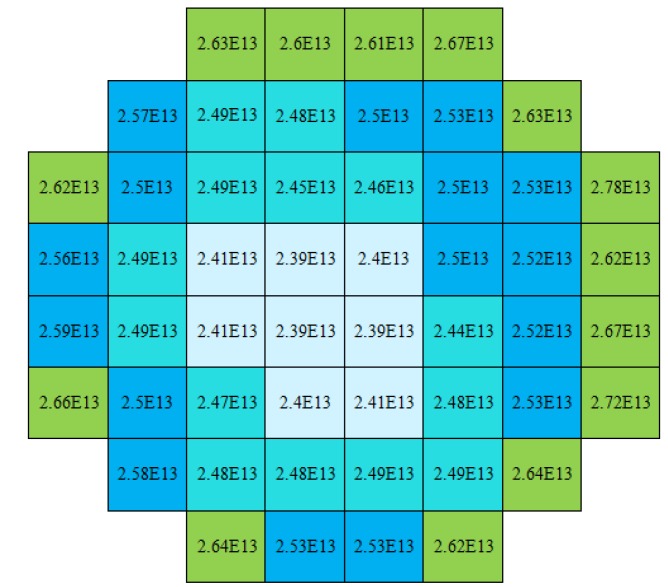
Over-the-wafer doping density in atoms-cm^−3^ measured by CV-profiling on 52 diodes with 7.6-mm^2^ anode-area at 3 V reverse bias.

The over-the-wafer doping uniformity is evaluated for the 40-μm-thick epi-layers from 52 measurement points over the 4-inch wafer. The circular spread of doping visible in Figure 14 correlates with the rotation of the wafer on the susceptor during deposition, and the drop in doping density towards the wafer center is in accordance with dopant source depletion as is common in epitaxial processes. The values presented in the figure are extracted for a depth of 25 μm from the wafer surface that corresponds to ~3 V reverse bias. The doping uniformity over the wafer is calculated as a standard deviation over the average doping (σ/average) to be 3.6%.

## 5. Implementation of As-Doped Epi-Layer on SEM Backscattered-Electron Detectors

**Figure 15:**
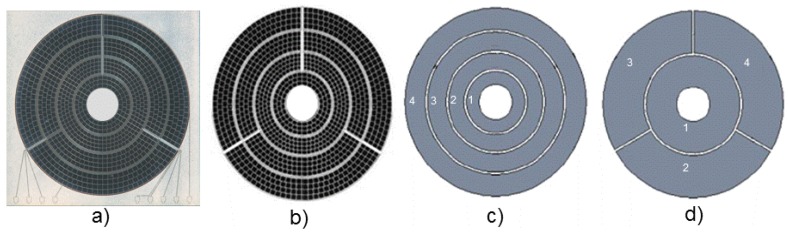
(**a**) Photograph of the PureB detector; (**b**) The electron detector consists of eight segments that can be used in two modes by combining the signal of segments into the (**c**) CBS—Concentric-Back-Scattered and (**d**) ABS—Angular-Back-Scatter mode.

Modern solid-state electron detectors require a high degree of design freedom and flexibility. This includes the segmentation of the photosensitive surface of a large-area detector into a number of detector segments that can function independently or in groups as the application dictates. The motivation for this is to detect not only the amount of incident particles, but also their position and the angular distribution. An example of an 8-segment layout is given in Figure 15, and it is demonstrated how the individual segments can be grouped in Figures 15(c,d).

**Figure 16:**
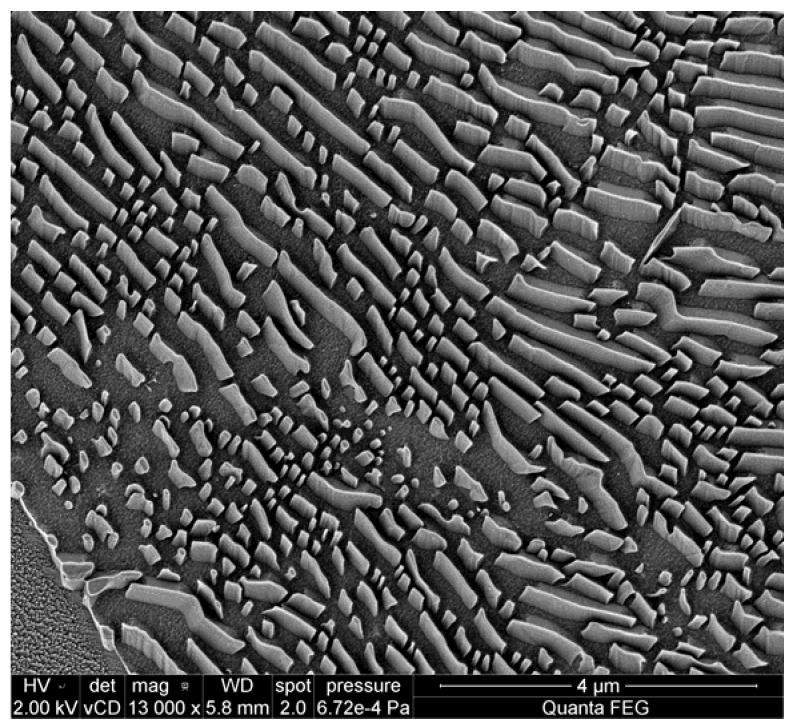
SEM image of a metal surface taken at 2 kV with the Concentric-Backscattered configuration (Figure 15(c)) of a PureB electron detector [1].

When designing detectors for SEM systems, there is a trade-off between the packing density of the segments and the doping of the epi, *i.e.*, the capacitance. In this work a separation between segments of 100 μm for the 40-μm-thick epi-layers was achieved together with a capacitance of 30 pF for the 7.6 mm^2^ detector area. Thus the <5 pF/cm^2^ specification was reached. An example of imaging with the segmented layout is given in Figure 16, where the Concentric-Back-Scattered mode illustrated in Figure 15(c) is used to enhance the topographical contrast of the inspected sample.

## 6. Conclusions

A method of growing high-resistivity, high-quality, thick epi-layers using the segregation behavior of arsenic for doping has been discussed. The motivation for the work stems from common requirements placed on solid-state charged-particle detectors in terms of low capacitance and resistance values, and a high degree of flexibility in the pixelation of the photosensitive surface. To achieve wide depletion regions and low series resistance through the substrate, epi-layers are grown on 2–5 Ωcm substrates, to a thicknesses of 5 μm, 10 μm, 20 μm, and 40 μm, and the doping levels are successfully reduced down to 5 × 10^11^ cm^−3^. The doping level and the profile are discussed in terms of three main parameters: the arsenic dopant source concentration (seed layer), the growth cycles, and the total thickness of the layer. The concentration of the initially grown seed layer, that is an As-doped Si layer of 300 nm thickness and 10^16^ or 10^17^ cm^−3^As concentration, clearly controls the overall doping level of the epi layer. Second, growing thick epi-layers with interruptions in growth sequence, followed by a 1,100 °C baking step before continuing the growth promotes As segregation to the surface. This gives a slight increase in doping around the place of growth interruption which can be beneficial for preventing the doping level from becoming too light and therefore susceptible to counter-doping from contamination in the epi chamber. Several layer thicknesses have been fabricated and studied with respect to capacitance and the resistance values of the diodes fabricated on such substrates. For 7.6 mm^2^ diode areas with 40-μm-thick epi-layers, capacitance values as low as 30 pF have been achieved, together with a diode series resistance of 90 Ω at 3 V reverse bias. A low dark current level is achieved and the presence of small concentrations of stacking faults does not degrade the dark current or capacitance of the diodes. The light n-doping is uniform within 3.6% over the wafer. The obtained characteristics have made it possible to enhance the performance of SEM systems equipped with these detectors with respect to, for example, the material and topographical contrasts of the acquired images.
